# Combined enteroscopy and choledochoscopy for recanalization of complete bilioenteric anastomotic occlusion after pancreaticoduodenectomy: a novel minimally invasive approach

**DOI:** 10.1055/a-2976-5877

**Published:** 2026-10-27

**Authors:** Xin Huang, Wenjuan Zhang, Changqing Zhong, Kai Lin, Bin Liang, Shaojie Zheng, Lianyong Li

**Affiliations:** 1 Department of Gastroenterology 739621 The Ninth Medical Center of Chinese PLA General HospitalBeijingBeijingChina; 2 Department of Hepatobiliary and Pancreatic Surgery 739621 The Ninth Medical Center of Chinese PLA General HospitalBeijingBeijingChina

Bilioenteric anastomotic stricture represents one of the postoperative complications of pancreaticoduodenectomy and is routinely managed with enteroscopy-assisted endoscopic retrograde cholangiopancreatography (ERCP) or percutaneous transhepatic biliary drainage (PTBD) in clinical practice. 1​ Complete anastomotic occlusion, by contrast, is rare and far more refractory than simple strictures. The inability to advance a guidewire across the occluded segment typically renders ERCP technically unfeasible, necessitating repeat surgical intervention.

A 68-year-old woman underwent pancreaticoduodenectomy for poorly differentiated pancreatic head adenocarcinoma. Five months postoperatively, the patient developed obstructive jaundice, and PTBD was performed for biliary decompression. Four weeks after PTBD placement, choledochoscopy via the PTBD sinus tract revealed complete occlusion of the bilioenteric anastomosis, and multiple attempts to advance a guidewire across the occlusion were unsuccessful ( Fig. 1 ). Oral double-balloon enteroscopy failed to visualize the anastomotic site; meticulous inspection revealed only a small, flat, mature scar.

**Fig. 1:**
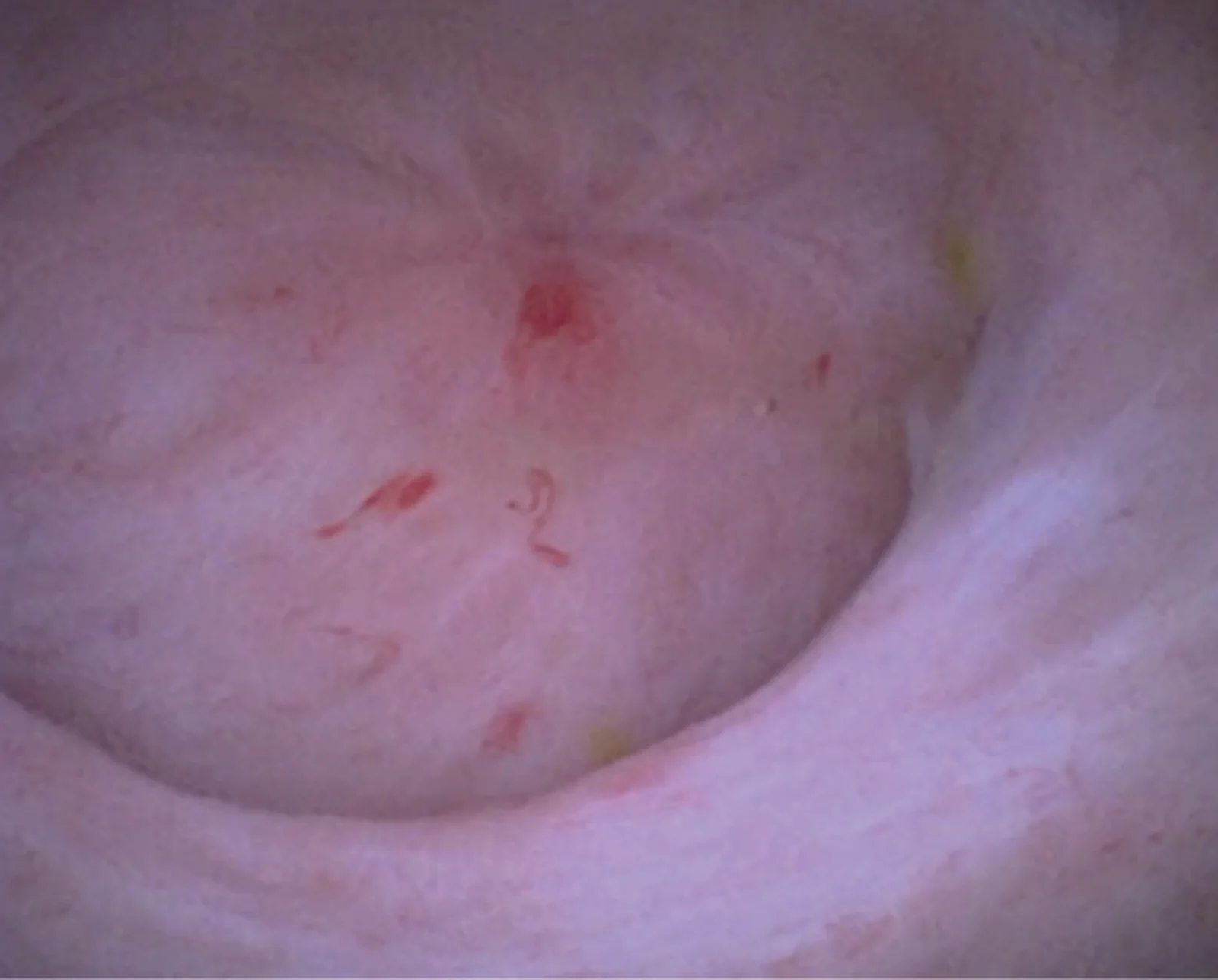
A 9-Fr choledochoscope was advanced through the PTBD sinus tract, revealing complete occlusion of the biliary anastomosis.

Activation of the choledochoscope yielded clear bidirectional red transillumination between the two endoscopes, and fluoroscopy verified that the scar corresponded to the obliterated anastomotic site ( Fig. 2 ). Under direct visualization and confirmation by choledochoscopy, enteroscopic needle puncture and mini-incision with a dual knife are performed ( Fig. 3 ). A guidewire was passed via the choledochoscope, and serial tract dilation was performed. Dual 7-Fr biliary stents were deployed with fluoroscopic monitoring ( Fig. 4 ). Cholangiography via the PTBD access tract showed unobstructed passage of contrast medium into the intestinal lumen, and no extravasation was noted ( Fig. 5 , Video 1 ).

**Fig. 2:**
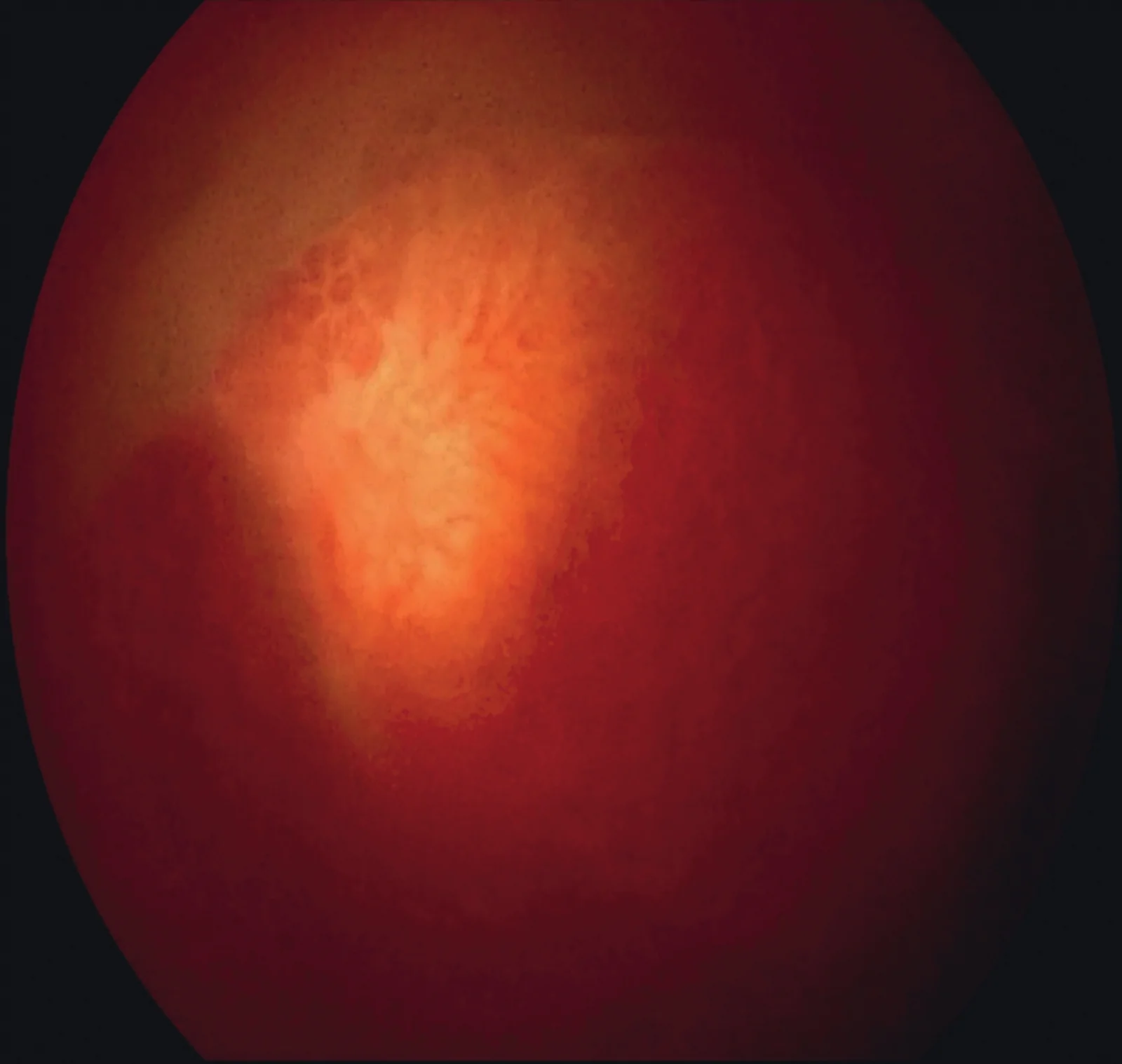
The light source of the choledochoscope was positioned against the center of the scar, and a distinct red light signal was visualized via the enteroscope.

**Fig. 3:**
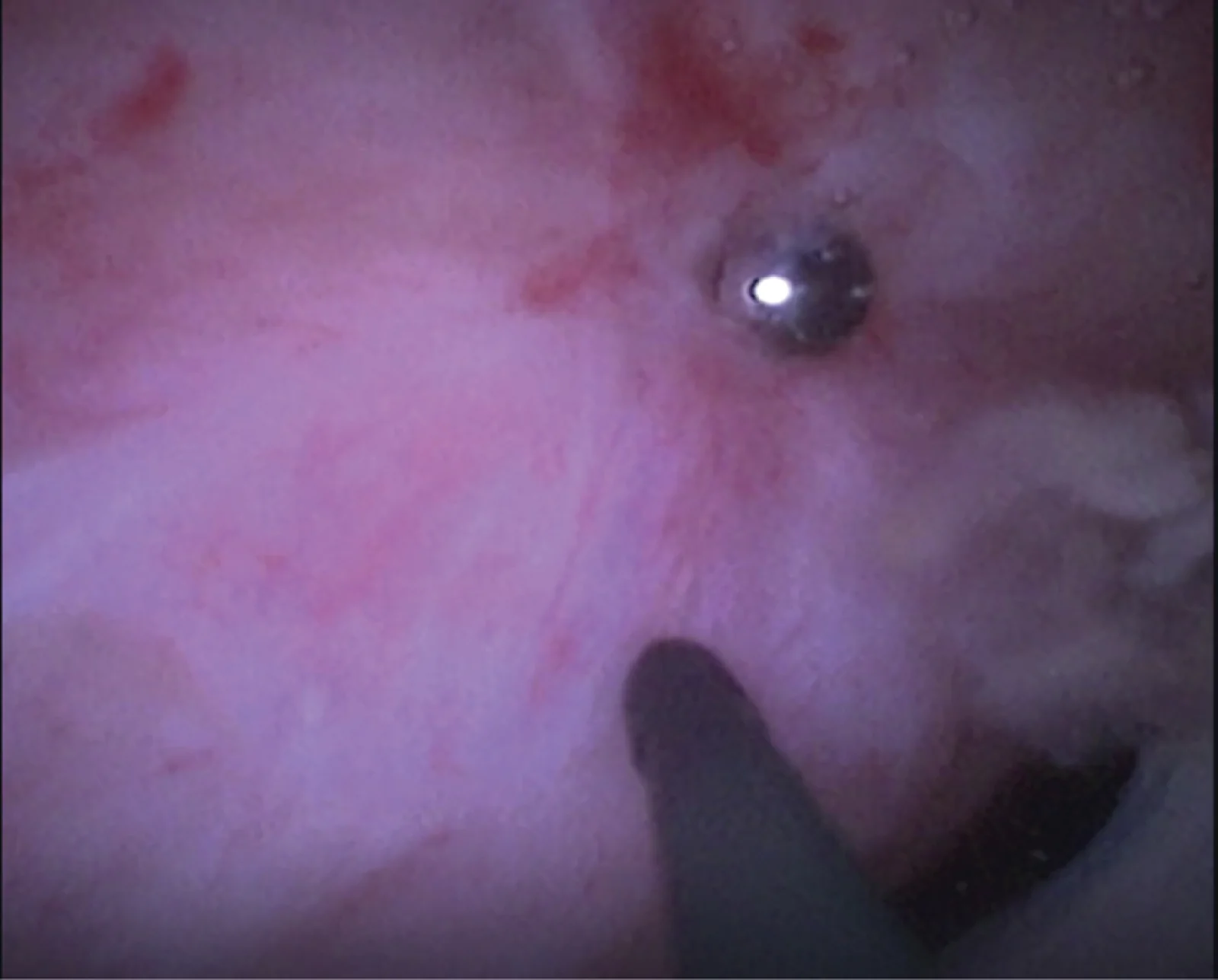
Enteroscopic needle puncture and dual knife mini-incision under direct choledochoscopic confirmation.

**Fig. 4:**
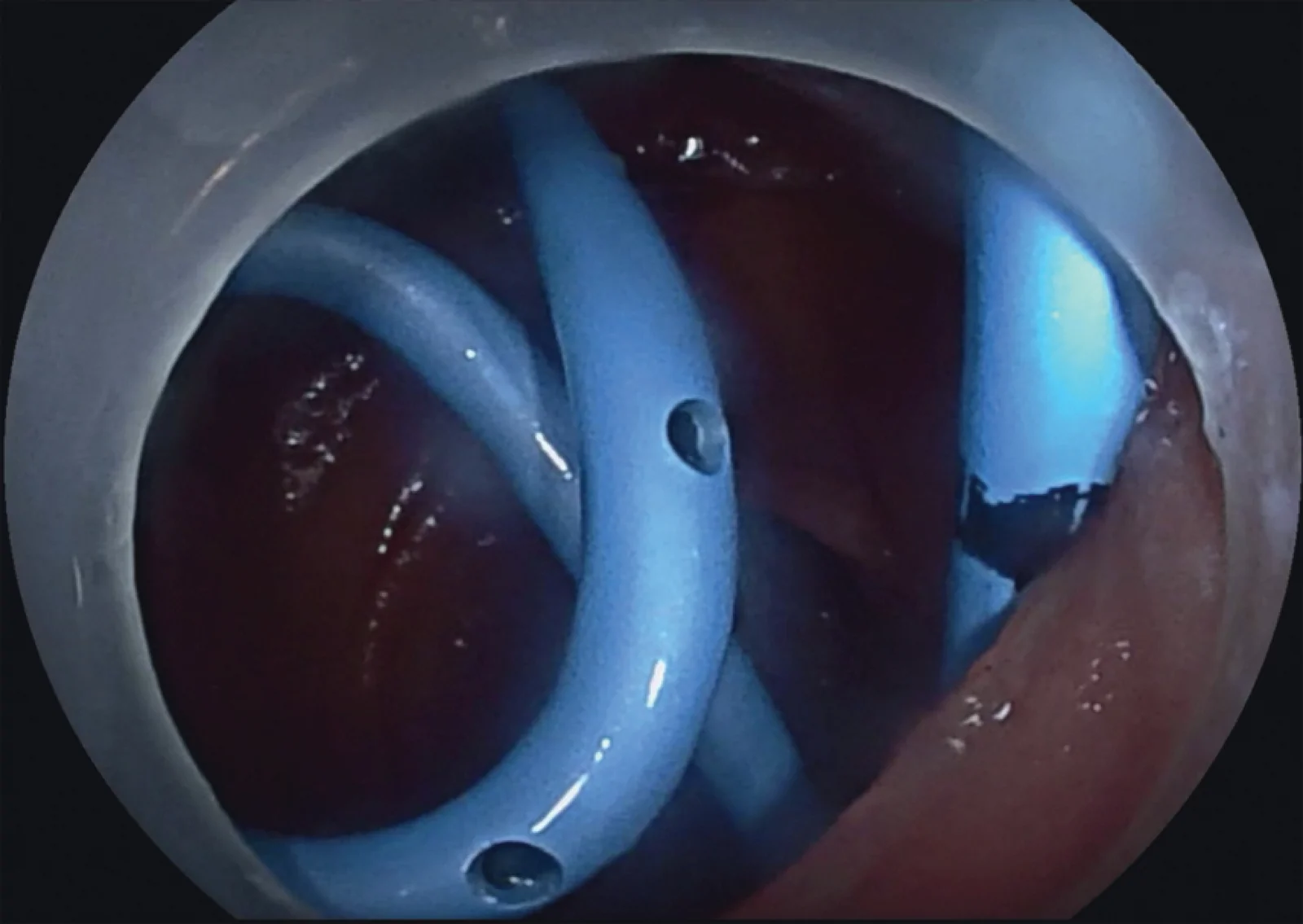
Two 7-Fr biliary stents are deployed over retained guidewires for biliary drainage.

**Fig. 5:**
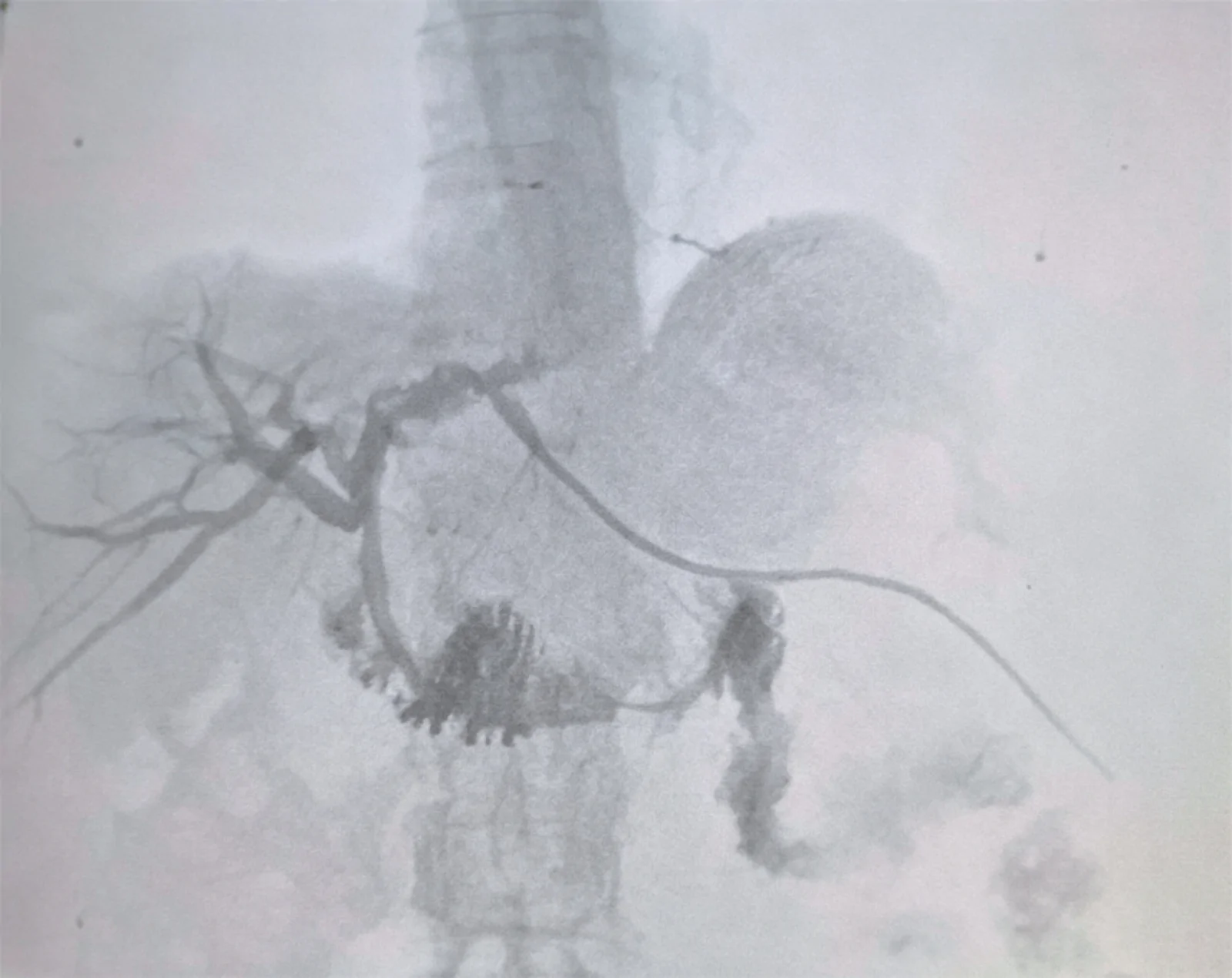
Cholangiography via the PTBD catheter shows contrast flows freely into intestine without extravasation.

**Video 1** Combined enteroscopy and choledochoscopy for recanalization of complete bilioenteric anastomotic occlusion, with transillumination-guided needle puncture, incision, dilation, and biliary stent deployment. URI: .


The patient tolerated the dual-endoscopic procedure uneventfully without immediate perioperative complications and was discharged on postoperative day 3. This case confirms that the combined endoscopic approach is an effective minimally invasive option to recanalize completely obliterated bilioenteric anastomoses after pancreaticoduodenectomy, avoiding re-laparotomy; additionally, it lowers perforation risks caused by blind incision with single-endoscope manipulation. 2​

Endoscopy_UCTN_Code_TTT_1AR_2AL

## Declaration of GenAI Use

The authors confirm that no generative artificial intelligence was used in the preparation of this manuscript.
